# Supplementary material for: Fitness benefits of a synonymous substitution in an ancient EF-Tu gene depend on the genetic background
Source: J Bacteriol. 2024 Jan 30;206(2):e00329-23. doi: 10.1128/jb.00329-23 (PMC10882980; doi:10.1128/jb.00329-23)
Supplement: Supplemental material — Tables S1 to S3; Figures S1 to S4. [file jb.00329-23-s0001.docx]

**Supplementary Table 1**: Strains and genetic markers used in this study.

| **Strain** | **Species, genotype, or characteristics** |
| --- | --- |
| REL606 (WT) | *Escherichia coli*, F-, tsx-467(Am), araA230, lon-, rpsL227(strR), hsdR-, [mal+](LamS) |
| REL607 | REL606 *araA* D92G (GAC→GGC) |
| *∆tufA* | REL606 *∆tufA* |
| Ancestor | REL606 ∆*tufA*, *tufB*::*AnEF* |
| Evolved | REL606 ∆*tufA*, *tufB*::*AnEF* V15V (GTC→GTT), *mdrA* T461P (ACC→CCC), *ydfI* Insertion +9 bp, *pykF* T278P (ACC→CCC), *ydjN* Δ24 bp, *yfaS* R900C (CGT→TGT) |
| CH3998 | MG1655 yfaH::[TP22-amilCP_opt-kan-sacB-T0] |
| CH1940 | MG1655 / pSIM5-tet |
| CH6523 | REL606 ∆*tufA, tufB*::*AnEF,* TP22-amilCP_opt-kan-sacB-T0 in intergenic region between *rpoC* and *yjaZ* |
| CH6557 | REL606 ∆*tufA, tufB*::*AnEF_C45T_,* evolved for 3000 generations, TP22-amilCP_opt-kan-sacB-T0 in intergenic region between *rpoC* and *yjaZ* |
| CH6556 | REL606 ∆*tufA, tufB*::*AnEF_,_* rest of genome evolved for 3000 generations. TP22-amilCP_opt-kan-sacB-T0 in intergenic region between *rpoC* and *yjaZ* |
| CH6585 | REL606 ∆*tufA, tufB*::*AnEF_C45T,_* rest of genome ancestral*.* TP22-amilCP_opt-kan-sacB-T0 in intergenic region between *rpoC* and *yjaZ* |
| g500 | REL606 ∆*tufA*, *tufB*::*AnEF*, evolved 500 generations (MB+2 500) |
| g1000 | REL606 ∆*tufA*, *tufB*::*AnEF* , evolved 1000 generations (MB+2 1000) |
| g1500 | REL606 ∆*tufA*, *tufB*::*AnEF* , evolved 1500 generations (MB+2 1500) |
| g2000 | REL606 ∆*tufA*, *tufB*::*AnEF* , evolved 2000 generations (MB+2 2000) |
| g2500 | REL606 ∆*tufA*, *tufB*::*AnEF* , evolved 2500 generations (MB+2 2500) |
| g3000 | REL606 ∆*tufA*, *tufB*::*AnEF* , evolved 3000 generations (MB+2 3000) |
| Evolved (AnEF_C45T_) | REL606 ∆*tufA*, *tufB*::*AnEF*, evolved 3000 generations, *AnEF* C45T (GTC→GTT) |
| Evolved (AnEF_T45C_) | REL606 ∆*tufA*, *tufB*::*AnEF*, evolved 3000 generations, *AnEF* T45C *(*GTT→GTC) |
| Ancestor (AnEF_C45T)_ | REL606 ∆*tufA*, *tufB*::*AnEF* C45T (GTC→GTT) |

**Supplementary Table 2**: Oligonucleotide primers used in this study.

| **Oligo name** | **Sequence (5’ to 3’)** |
| --- | --- |
| 606_ksacBblue_linketufB_fw | AGGGAAAGAGCATTTGTCAGAATATTTAAGGAATTTCTGAATCAAAGGGAAAACTGTCCA |
| 606_ksacBblue_linketufB_rv | GTTTCAGGATATTAGTCATCTCTACATTGATTATGAGTATAAAATGAGACGTTGATCGGC |
| 606_rpoC_out_fw | GTTATCGTGGGTCGTCTG |
| 606_yjaZ_out_rv | TGTTTTTCTTCGTTCGTCTG |
| kan_out | GTCATAGCCGAATAGCCTCTCCAC |
| sacB_out | GCTGTACCTCAAGCGAAAGG |
| tufB_coli_fw | TTCTTTTCTCCTCCCTGT |
| tufB_coli_rv | GGCAAACCAAATCGAAAC |
| qPCR_tufB_fwd | AACCGCACGTTAACGTCGGT |
| qPCR_tufB_rev | GCCAGTACGGTAGTGATTGCAGC |
| qPCR_rpoB_fwd | TAAGGTAACGCCGAAAGGTG |
| qPCR_rpoB_rev | CAGAGGCTTTCTCACCGAAG |

**Supplementary Table 3** – Distribution of fixed mutations across 3000 generations in experimentally evolved *E. coli* REL606 (*tufA∆*, *tufB*::*AnEF*).

| **gene** | **mutation** | **g0** | **g500** | **g1000** | **g1500** | **g2000** | **g2500** | **g3000** |
| --- | --- | --- | --- | --- | --- | --- | --- | --- |
| *ydjN* | Δ24 bp | 0% | 100% | 100% | 100% | 100% | 100% | 100% |
| *fadA* | Insertion +3 bp | 0% | 100% | 100% | 100% | 100% | 100% | 100% |
| *rbsD*–[*rbsR*] | Δ5,379 bp | 0% | 100% | 100% | 100% | 100% | 100% | 100% |
| *mrdA* | T461P (ACC→CCC) | 0% | 90.2% | 100% | 100% | 100% | 100% | 100% |
| *ydfI* | Insertion +9 bp | 0% | 94.2% | 100% | 100% | 100% | 100% | 100% |
| *yfaS* | R900C (CGT→TGT) | 0% | 94.3% | 100% | 100% | 100% | 100% | 100% |
| *pykF* | T278P (ACC→CCC) | 0% | 0% | 21% | 15.8% | 60.2% | 100% | 100% |
| *AnEF* | V15V (GTC→GTT) | 0% | 0% | 0% | 0% | 0% | 100% | 100% |


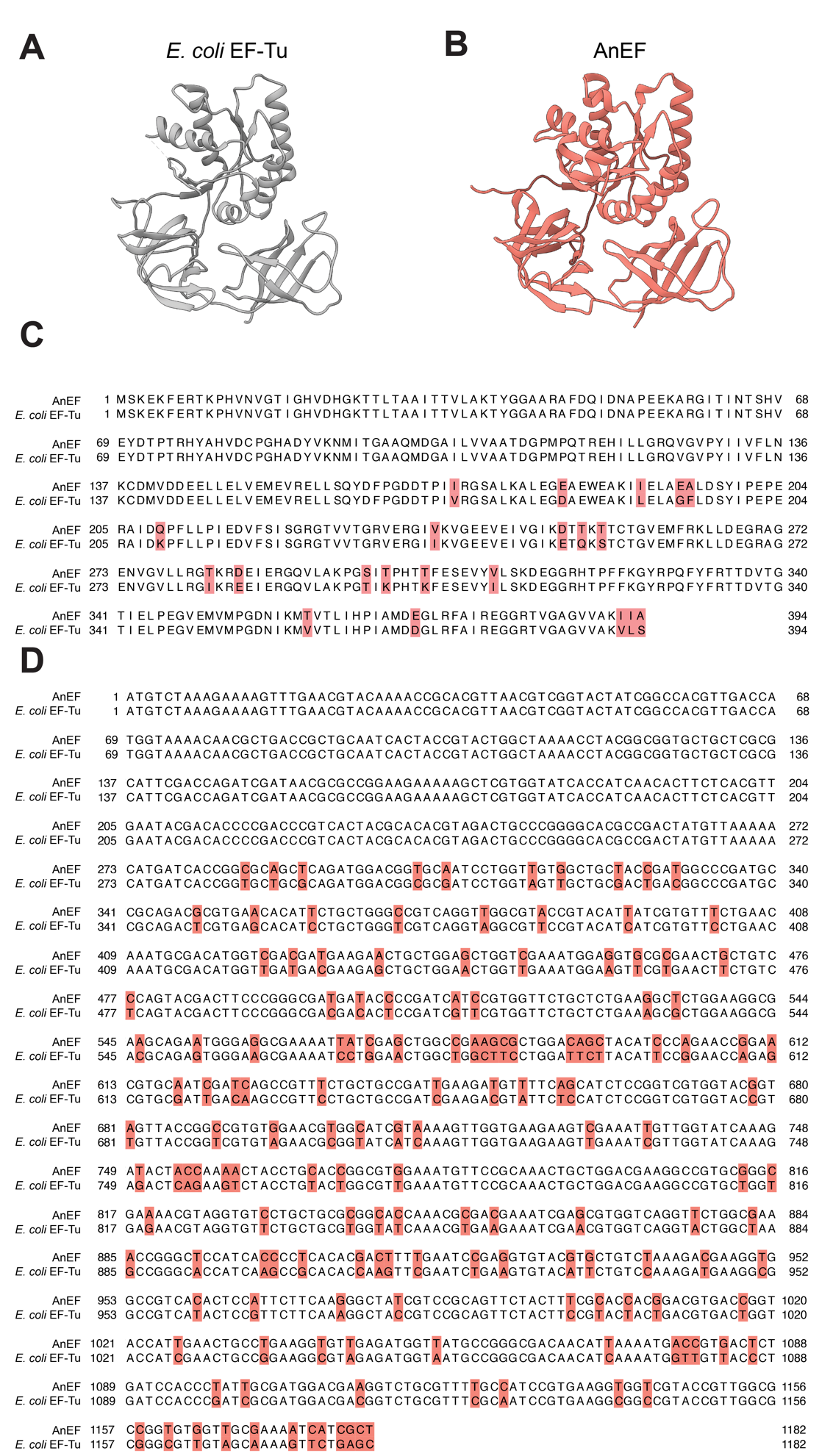


**Supplementary Figure 1**: Sequences and structural comparison. **(A)** Crystallized structure of *E. coli* EF-Tu (PDB ID: 5AFI) **(B)** Modeled structure of ancient EF-Tu (AnEF) protein (Methods). **(C)** Alignment of *E. coli* EF-Tu and AnEF protein sequences using MAFFT. Differences in sequence highlighted in red. Protein sequence identity 94.67%. **(D)** Alignment of *E. coli* *tufB* and *anEF* nucleotide sequences using MAFFT. Differences in sequence highlighted in red. Nucleotide sequence identity 87%.


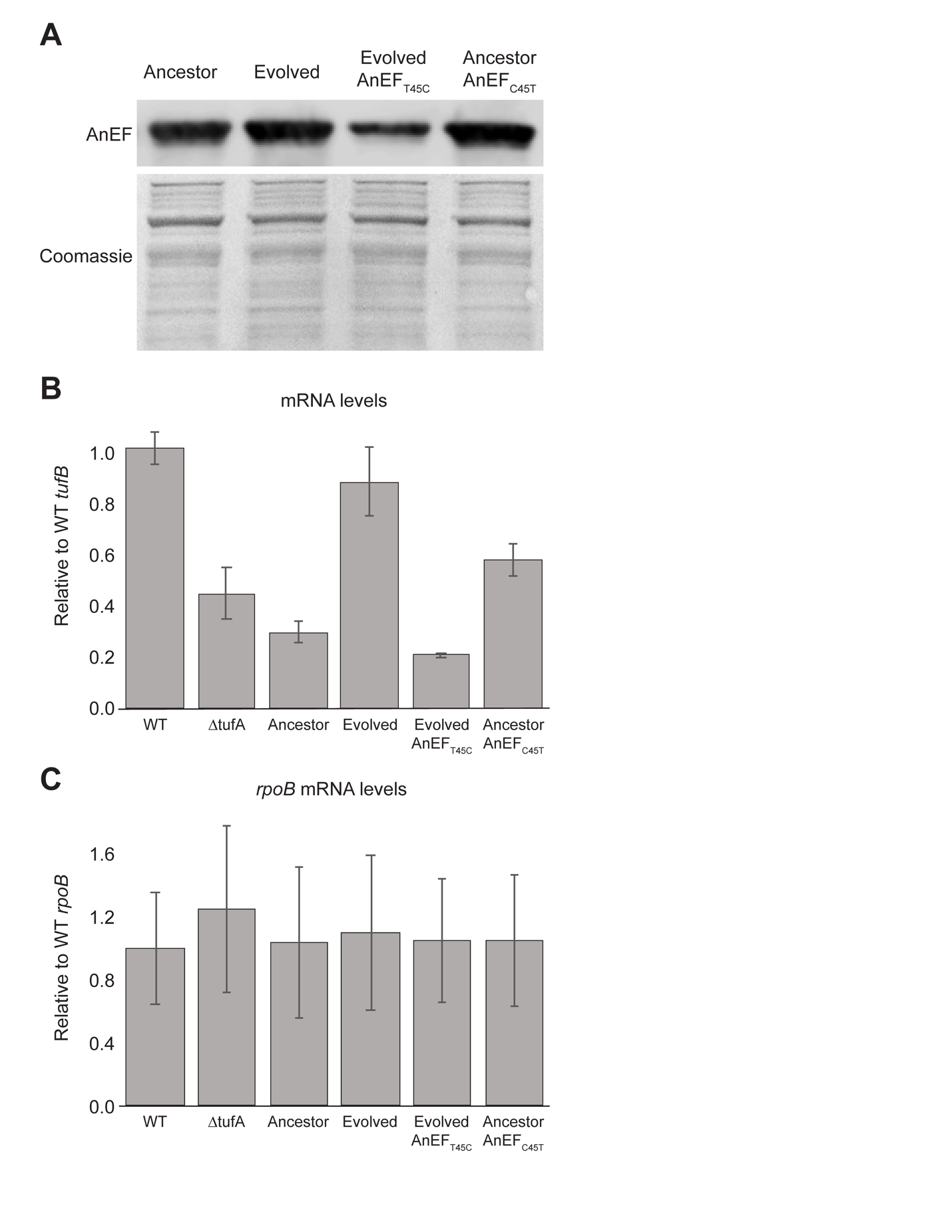


**Supplementary Figure 2**: AnEF protein and RNA levels. (A) AnEF protein levels represented via immunoblot. Total protein was stained using Coomassie. (n = 9) (B) qPCR quantification of *AnEF* and *tufB* mRNA, including REL606 (WT) and REL606 ∆*tufA*. (n = 3) (C) qPCR quantification of control *rpoB* mRNA.


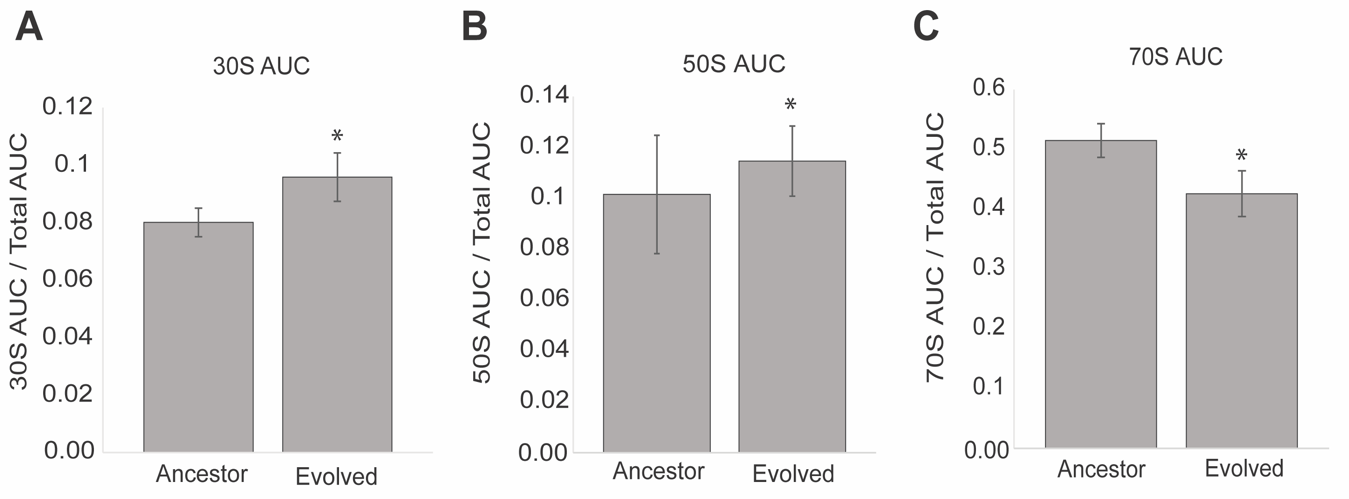


**Supplementary Figure 3**: Polysome profile area under the curve (AUC) quantification. Comparing the ribosomal components in ancestor vs. evolved strains of the (A) 30S ribosomal subunits, (B) 50S ribosomal subunit, (C) 70S monosomes.


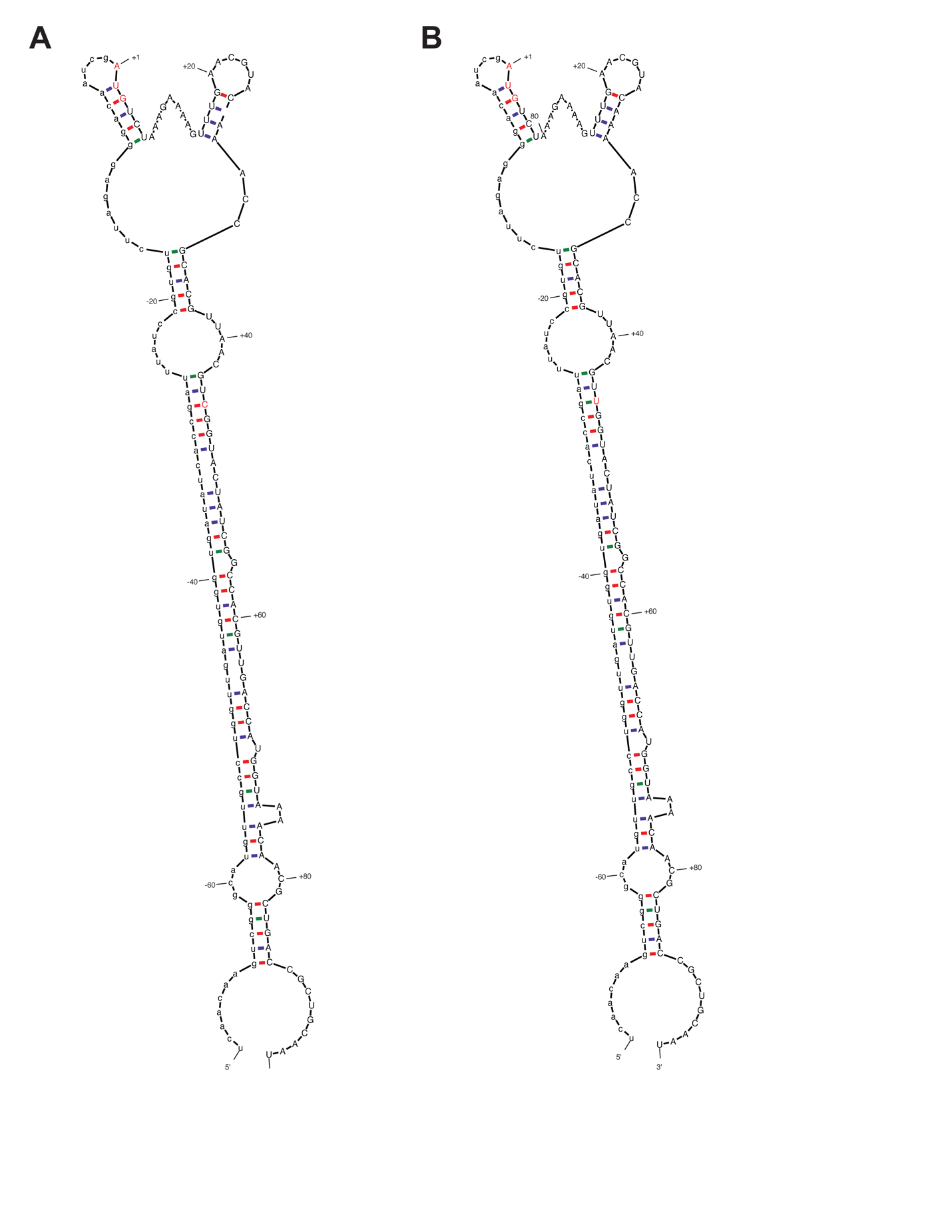


**Supplementary Figure 4**: mFold predicted secondary structures in the early region (from -73 to +96) of the (A) *E. coli* *tufB* and (B) *anEF_C45T_* mRNA.
